# Tetra-μ-methacrylato-κ^8^ *O*:*O*′-bis­[(pyri­din-2-amine-κ*N* ^1^)copper(II)]

**DOI:** 10.1107/S160053680901352X

**Published:** 2009-04-18

**Authors:** Xin-Yan Zhang

**Affiliations:** a Li Shui Vocational and Technical College, Lishui, Zhejiang 323000, People’s Republic of China

## Abstract

In the title carboxyl­ate-bridged binuclear copper complex, [Cu_2_(C_4_H_5_O_2_)_4_(C_5_H_6_N_2_)_2_], each Cu^II^ ion has a distorted square-based pyramidal environment formed by one N and four O atoms. The asymmetric unit contains two halves of two centrosymmetric mol­ecules, with Cu⋯Cu separations of 2.6498 (8) and 2.6528 (8) Å.

## Related literature

For the crystal structures of related binuclear complexes, see: Du *et al.* (2002 ▶); Wu & Wang (2004 ▶).
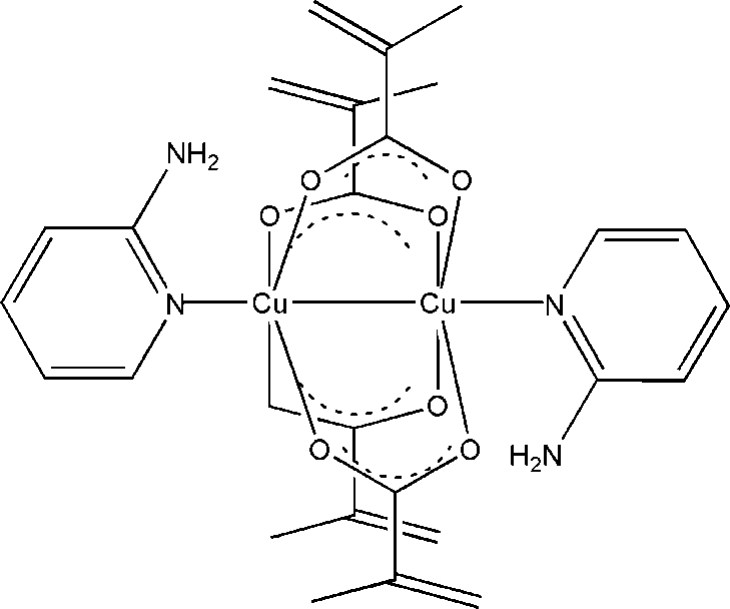


## Experimental

### 

#### Crystal data

[Cu_2_(C_4_H_5_O_2_)_4_(C_5_H_6_N_2_)_2_]
*M* *_r_* = 655.64
Monoclinic, 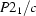

*a* = 16.8591 (15) Å
*b* = 12.1185 (11) Å
*c* = 16.5980 (15) Å
β = 117.458 (2)°
*V* = 3009.1 (5) Å^3^
*Z* = 4
Mo *K*α radiation
μ = 1.47 mm^−1^
*T* = 298 K
0.28 × 0.20 × 0.13 mm

#### Data collection

Bruker APEXII area-detector diffractometer
Absorption correction: multi-scan (*SADABS*; Sheldrick, 2004 ▶) *T*_min_ = 0.685, *T*_max_ = 0.832
15356 measured reflections
5539 independent reflections
3542 reflections with *I* > 2σ(*I*)
*R*_int_ = 0.099

#### Refinement

*R*[*F* ^2^ > 2σ(*F* ^2^)] = 0.041
*wR*(*F* ^2^) = 0.084
*S* = 0.88
5539 reflections
365 parameters
H-atom parameters constrained
Δρ_max_ = 0.50 e Å^−3^
Δρ_min_ = −0.35 e Å^−3^

### 

Data collection: *APEX2* (Bruker, 2004 ▶); cell refinement: *SAINT* (Bruker, 2004 ▶); data reduction: *SAINT*; program(s) used to solve structure: *SHELXS97* (Sheldrick, 2008 ▶); program(s) used to refine structure: *SHELXL97* (Sheldrick, 2008 ▶); molecular graphics: *ORTEPIII* (Burnett & Johnson, 1996 ▶) and *ORTEP-3 for Windows* (Farrugia, 1997 ▶); software used to prepare material for publication: *SHELXL97*.

## Supplementary Material

Crystal structure: contains datablocks I, global. DOI: 10.1107/S160053680901352X/cv2539sup1.cif

Structure factors: contains datablocks I. DOI: 10.1107/S160053680901352X/cv2539Isup2.hkl

Additional supplementary materials:  crystallographic information; 3D view; checkCIF report

## Figures and Tables

**Table 1:** Hydrogen-bond geometry (Å, °)

*D*—H⋯*A*	*D*—H	H⋯*A*	*D*⋯*A*	*D*—H⋯*A*
N2—H2*A*⋯O5^i^	0.86	2.23	2.963 (4)	144
N4—H4*A*⋯O4	0.86	2.30	3.051 (4)	145

Symmetry code: (i) 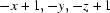.

**Fig. 1.**
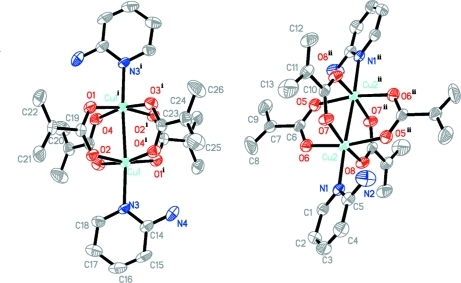
A portion of the title cystal structure showing two independent dinuclear complexes with the atom-labeling scheme [symmetry codes: (i) -x+2, -y+1, -z+2; (ii) -x+1, -y, -z+1]. Displacement ellipsoids are shown at the 30% probability level.

## supplementary crystallographic information

### Comment

In continuation of structural study of paddle-wheel copper(II) carboxylate compounds (Wu & Wang, 2004; Du *et al.*,  2002), we report here the crystal structure of the title complex, (I).

The title compound (Figure 1) contains two independent dinuclear cage Cu^II^ complexes, each with inversion symmetry. In each dimer, two Cu^II^ atoms are connected by four carboxylate groups from methacrylate ends, forming a cage structure, and the N atoms from the pyridin-2-amine ligands are binded  to Cu^II^ centers in the terminal positions, which is very similar the [Cu_2_{CH_2_C(CH_3_)COO}_4_(C_5_H_5_N)_2_] compound (Wu & Wang, 2004). In the title compound, the amino groups are involved in hydrogen-bonded interactions with carboxylate groups (Table 1). The Cu···Cu separations of 2.6498 (8) and 2.6528 (8) Å are a little longer than those in [Cu_2_{CH_2_C(CH_3_)COO}_4_(C_5_H_5_N)_2_] (Wu & Wang, 2004), that may be attributed to the steric effect from amino group in the assembling process.

### Experimental

Pyridin-2-amine (0.042 g, 0.28 mmol), [Cu_2_{CH_2_C(CH_3_)COO}_4_.2H_2_O] (0.025 g, 0.13 mmol), were added distilled methanol(20 mL), the mixture was heated for ten hours under reflux. during the process stirring and influx were required. The resultant was kept at room temperature, two days later single crystals suitable for X-ray diffraction measurement were obtained.

### Refinement

All H atoms were fixed geometrically (C—H = 0.93-0.96 Å, N—H = 0.86 Å) and treated as riding with  U_iso_(H) = 1.2-1.5U_eq_ of the parent atom.

### Crystal data

**Table:**

[Cu_2_(C_4_H_5_O_2_)_4_(C_5_H_6_N_2_)_2_]	*F*(000) = 1352
*M**_r_* = 655.64	*D*_x_ = 1.447 Mg m^−^^3^
Monoclinic, *P*2_1_/*c*	Mo *K*α radiation, λ = 0.71073 Å
Hall symbol: -P 2ybc	Cell parameters from 5539 reflections
*a* = 16.8591 (15) Å	θ = 2.2–25.4°
*b* = 12.1185 (11) Å	µ = 1.47 mm^−^^1^
*c* = 16.5980 (15) Å	*T* = 298 K
β = 117.458 (2)°	Block, blue
*V* = 3009.1 (5) Å^3^	0.28 × 0.20 × 0.13 mm
*Z* = 4	

### Data collection

**Table:**

Bruker APEXII area-detector diffractometer	5539 independent reflections
Radiation source: fine-focus sealed tube	3542 reflections with *I* > 2σ(*I*)
graphite	*R*_int_ = 0.099
φ and ω scans	θ_max_ = 25.4°, θ_min_ = 2.2°
Absorption correction: multi-scan (*SADABS*; Sheldrick, 2004)	*h* = −20→20
*T*_min_ = 0.685, *T*_max_ = 0.832	*k* = −14→10
15356 measured reflections	*l* = −20→20

### Refinement

**Table:**

Refinement on *F*^2^	Primary atom site location: structure-invariant direct methods
Least-squares matrix: full	Secondary atom site location: difference Fourier map
*R*[*F*^2^ > 2σ(*F*^2^)] = 0.041	Hydrogen site location: inferred from neighbouring sites
*wR*(*F*^2^) = 0.084	H-atom parameters constrained
*S* = 0.88	*w* = 1/[σ^2^(*F*_o_^2^) + (0.01*P*)^2^ + 0.272*P*] where *P* = (*F*_o_^2^ + 2*F*_c_^2^)/3
5539 reflections	(Δ/σ)_max_ = 0.001
365 parameters	Δρ_max_ = 0.50 e Å^−^^3^
0 restraints	Δρ_min_ = −0.35 e Å^−^^3^

### Special details

**Table:**

Geometry. All esds (except the esd in the dihedral angle between two l.s. planes) are estimated using the full covariance matrix. The cell esds are taken into account individually in the estimation of esds in distances, angles and torsion angles; correlations between esds in cell parameters are only used when they are defined by crystal symmetry. An approximate (isotropic) treatment of cell esds is used for estimating esds involving l.s. planes.
Refinement. Refinement of F^2^ against ALL reflections. The weighted R-factor wR and goodness of fit S are based on F^2^, conventional R-factors R are based on F, with F set to zero for negative F^2^. The threshold expression of F^2^ > 2sigma(F^2^) is used only for calculating R-factors(gt) etc. and is not relevant to the choice of reflections for refinement. R-factors based on F^2^ are statistically about twice as large as those based on F, and R- factors based on ALL data will be even larger.

### Fractional atomic coordinates and isotropic or equivalent isotropic displacement parameters (Å^2^)

**Table:**

	*x*	*y*	*z*	*U*_iso_*/*U*_eq_	
Cu2	0.45444 (2)	0.09186 (3)	0.49285 (2)	0.04205 (13)	
O6	0.57214 (14)	0.1643 (2)	0.55362 (14)	0.0594 (7)	
O5	0.64848 (13)	0.0105 (2)	0.56541 (14)	0.0568 (6)	
O7	0.46883 (14)	0.0508 (2)	0.61306 (13)	0.0559 (6)	
O8	0.45651 (13)	0.1057 (2)	0.37567 (13)	0.0531 (6)	
N1	0.37557 (15)	0.2416 (2)	0.47289 (15)	0.0420 (7)	
N2	0.26243 (18)	0.1607 (3)	0.4918 (2)	0.0837 (11)	
H2A	0.2881	0.0979	0.4969	0.100*	
H2B	0.2133	0.1647	0.4955	0.100*	
C1	0.4104 (2)	0.3339 (3)	0.45853 (19)	0.0498 (9)	
H1	0.4633	0.3273	0.4542	0.060*	
C2	0.3754 (2)	0.4363 (3)	0.4498 (2)	0.0637 (10)	
H2	0.4029	0.4976	0.4397	0.076*	
C3	0.2970 (3)	0.4456 (4)	0.4565 (2)	0.0739 (12)	
H3	0.2711	0.5146	0.4520	0.089*	
C4	0.2579 (2)	0.3550 (4)	0.4694 (2)	0.0671 (11)	
H4	0.2043	0.3606	0.4725	0.081*	
C5	0.2991 (2)	0.2524 (3)	0.4781 (2)	0.0511 (9)	
C6	0.6433 (2)	0.1113 (3)	0.5791 (2)	0.0491 (9)	
C7	0.7293 (2)	0.1718 (4)	0.6338 (2)	0.0638 (11)	
C8	0.7244 (3)	0.2751 (4)	0.6645 (3)	0.123 (2)	
H5	0.7764	0.3150	0.6986	0.148*	
H6	0.6691	0.3054	0.6514	0.148*	
C9	0.8112 (2)	0.1162 (4)	0.6499 (3)	0.0890 (14)	
H7	0.8151	0.0472	0.6799	0.134*	
H8	0.8614	0.1616	0.6875	0.134*	
H9	0.8114	0.1029	0.5930	0.134*	
C10	0.5107 (2)	−0.0342 (3)	0.6544 (2)	0.0459 (9)	
C11	0.5223 (2)	−0.0509 (4)	0.7493 (2)	0.0596 (10)	
C12	0.5509 (2)	−0.1495 (4)	0.7904 (2)	0.0842 (13)	
H12A	0.5571	−0.1610	0.8484	0.101*	
H12B	0.5645	−0.2058	0.7607	0.101*	
C13	0.5006 (3)	0.0384 (5)	0.7901 (3)	0.1203 (19)	
H13A	0.5050	0.0153	0.8474	0.180*	
H13B	0.4407	0.0623	0.7511	0.180*	
H13C	0.5413	0.0982	0.7997	0.180*	
Cu1	0.95058 (2)	0.58684 (3)	0.95769 (2)	0.04257 (13)	
O2	0.95450 (14)	0.6179 (2)	1.07602 (13)	0.0538 (6)	
O1	1.03977 (14)	0.4737 (2)	1.14736 (13)	0.0557 (6)	
O4	0.84923 (13)	0.4861 (2)	0.92871 (14)	0.0580 (6)	
O3	1.06707 (14)	0.6594 (2)	1.00035 (15)	0.0581 (6)	
N3	0.87554 (16)	0.7370 (2)	0.89766 (16)	0.0459 (7)	
N4	0.7646 (2)	0.6533 (3)	0.7748 (2)	0.1019 (13)	
H4A	0.7880	0.5907	0.7981	0.122*	
H4B	0.7171	0.6557	0.7233	0.122*	
C14	0.8020 (2)	0.7465 (4)	0.8181 (2)	0.0595 (10)	
C15	0.7644 (3)	0.8481 (4)	0.7818 (3)	0.0766 (13)	
H14	0.7130	0.8525	0.7264	0.092*	
C16	0.8035 (3)	0.9387 (4)	0.8278 (3)	0.0878 (14)	
H15	0.7798	1.0075	0.8040	0.105*	
C17	0.8792 (3)	0.9320 (4)	0.9108 (3)	0.0791 (12)	
H16	0.9065	0.9950	0.9440	0.095*	
C18	0.9116 (2)	0.8313 (3)	0.9417 (2)	0.0580 (10)	
H18	0.9626	0.8264	0.9974	0.070*	
C19	0.9986 (2)	0.5606 (3)	1.1453 (2)	0.0438 (8)	
C20	1.0036 (2)	0.6006 (3)	1.2327 (2)	0.0485 (9)	
C21	0.9650 (2)	0.7007 (4)	1.2336 (2)	0.0756 (12)	
H21A	0.9676	0.7271	1.2874	0.091*	
H21B	0.9364	0.7417	1.1805	0.091*	
C22	1.0486 (2)	0.5319 (4)	1.3096 (2)	0.0833 (13)	
H22A	1.0584	0.5718	1.3634	0.125*	
H22B	1.1051	0.5096	1.3139	0.125*	
H22C	1.0129	0.4678	1.3037	0.125*	
C23	0.8592 (2)	0.3867 (3)	0.9520 (2)	0.0484 (9)	
C24	0.7760 (2)	0.3184 (3)	0.9224 (2)	0.0632 (10)	
C25	0.6949 (3)	0.3669 (4)	0.8807 (3)	0.1101 (17)	
H25A	0.6434	0.3249	0.8636	0.132*	
H25B	0.6904	0.4423	0.8691	0.132*	
C26	0.7873 (3)	0.2029 (4)	0.9421 (4)	0.1107 (17)	
H26A	0.7305	0.1706	0.9287	0.166*	
H26B	0.8280	0.1923	1.0052	0.166*	
H26C	0.8108	0.1683	0.9057	0.166*	

### Atomic displacement parameters (Å^2^)

**Table:**

	*U*^11^	*U*^22^	*U*^33^	*U*^12^	*U*^13^	*U*^23^
Cu2	0.0353 (2)	0.0501 (3)	0.0383 (2)	0.00534 (19)	0.01493 (17)	0.0075 (2)
O6	0.0419 (13)	0.0637 (18)	0.0629 (14)	−0.0030 (13)	0.0160 (12)	0.0034 (14)
O5	0.0347 (13)	0.0625 (18)	0.0670 (15)	0.0007 (13)	0.0182 (11)	0.0027 (14)
O7	0.0655 (15)	0.0628 (17)	0.0452 (13)	0.0154 (14)	0.0304 (12)	0.0141 (12)
O8	0.0555 (14)	0.0648 (17)	0.0423 (12)	0.0132 (13)	0.0253 (11)	0.0124 (12)
N1	0.0351 (14)	0.0552 (19)	0.0365 (13)	0.0051 (14)	0.0171 (12)	0.0022 (13)
N2	0.064 (2)	0.088 (3)	0.126 (3)	0.013 (2)	0.067 (2)	0.023 (2)
C1	0.0408 (19)	0.061 (3)	0.0449 (19)	0.0041 (19)	0.0176 (16)	−0.0011 (19)
C2	0.067 (3)	0.050 (3)	0.068 (2)	0.003 (2)	0.027 (2)	0.000 (2)
C3	0.070 (3)	0.066 (3)	0.075 (3)	0.023 (2)	0.025 (2)	−0.007 (2)
C4	0.056 (2)	0.083 (3)	0.069 (3)	0.015 (2)	0.035 (2)	−0.005 (2)
C5	0.047 (2)	0.066 (3)	0.0431 (18)	0.007 (2)	0.0234 (16)	−0.0004 (19)
C6	0.041 (2)	0.064 (3)	0.0389 (19)	−0.007 (2)	0.0156 (17)	0.0116 (19)
C7	0.046 (2)	0.070 (3)	0.063 (2)	−0.010 (2)	0.0150 (19)	0.016 (2)
C8	0.078 (3)	0.084 (4)	0.159 (5)	−0.031 (3)	0.013 (3)	−0.038 (4)
C9	0.054 (2)	0.119 (4)	0.080 (3)	−0.017 (3)	0.019 (2)	0.014 (3)
C10	0.0346 (19)	0.061 (3)	0.0382 (18)	−0.0059 (18)	0.0136 (15)	0.0036 (18)
C11	0.060 (2)	0.081 (3)	0.0368 (19)	0.004 (2)	0.0221 (17)	0.007 (2)
C12	0.104 (3)	0.099 (4)	0.051 (2)	0.012 (3)	0.037 (2)	0.023 (2)
C13	0.181 (5)	0.128 (5)	0.063 (3)	0.028 (4)	0.066 (3)	0.001 (3)
Cu1	0.0397 (2)	0.0478 (3)	0.0397 (2)	0.0095 (2)	0.01785 (18)	0.00255 (19)
O2	0.0624 (14)	0.0598 (16)	0.0409 (12)	0.0190 (13)	0.0254 (11)	0.0052 (12)
O1	0.0651 (15)	0.0638 (18)	0.0424 (12)	0.0192 (14)	0.0283 (11)	0.0022 (12)
O4	0.0377 (13)	0.0662 (18)	0.0614 (14)	0.0051 (13)	0.0154 (11)	0.0078 (14)
O3	0.0415 (13)	0.0556 (17)	0.0765 (15)	0.0017 (12)	0.0267 (12)	0.0023 (14)
N3	0.0377 (15)	0.056 (2)	0.0390 (14)	0.0134 (15)	0.0136 (12)	0.0087 (14)
N4	0.090 (3)	0.105 (3)	0.057 (2)	0.010 (2)	−0.0116 (18)	0.002 (2)
C14	0.052 (2)	0.074 (3)	0.050 (2)	0.014 (2)	0.0207 (19)	0.010 (2)
C15	0.065 (3)	0.099 (4)	0.057 (2)	0.036 (3)	0.020 (2)	0.030 (3)
C16	0.098 (4)	0.079 (4)	0.091 (3)	0.042 (3)	0.047 (3)	0.036 (3)
C17	0.092 (3)	0.059 (3)	0.097 (3)	0.021 (3)	0.054 (3)	0.015 (3)
C18	0.060 (2)	0.054 (3)	0.062 (2)	0.012 (2)	0.0304 (19)	0.006 (2)
C19	0.0379 (18)	0.056 (2)	0.0425 (19)	−0.0068 (18)	0.0229 (16)	−0.0076 (18)
C20	0.0406 (19)	0.066 (3)	0.0394 (18)	−0.0094 (18)	0.0186 (15)	−0.0132 (18)
C21	0.092 (3)	0.084 (3)	0.056 (2)	0.011 (3)	0.038 (2)	−0.019 (2)
C22	0.091 (3)	0.106 (4)	0.052 (2)	0.001 (3)	0.032 (2)	−0.008 (3)
C23	0.047 (2)	0.054 (3)	0.047 (2)	−0.0013 (19)	0.0234 (17)	−0.0084 (18)
C24	0.052 (2)	0.065 (3)	0.071 (2)	−0.005 (2)	0.026 (2)	−0.015 (2)
C25	0.046 (2)	0.087 (4)	0.167 (5)	−0.001 (3)	0.023 (3)	−0.002 (4)
C26	0.083 (3)	0.067 (3)	0.174 (5)	−0.014 (3)	0.052 (3)	−0.008 (4)

### Geometric parameters (Å, °)

**Table:**

Cu2—O7	1.960 (2)	Cu1—O3	1.962 (2)
Cu2—O8	1.968 (2)	Cu1—O1^ii^	1.966 (2)
Cu2—O6	1.970 (2)	Cu1—O4	1.971 (2)
Cu2—O5^i^	1.985 (2)	Cu1—O2	1.971 (2)
Cu2—N1	2.183 (3)	Cu1—N3	2.178 (3)
Cu2—Cu2^i^	2.6528 (8)	Cu1—Cu1^ii^	2.6498 (8)
O6—C6	1.250 (4)	O2—C19	1.253 (4)
O5—C6	1.252 (4)	O1—C19	1.253 (4)
O5—Cu2^i^	1.985 (2)	O1—Cu1^ii^	1.966 (2)
O7—C10	1.258 (4)	O4—C23	1.252 (4)
O8—C10^i^	1.249 (4)	O3—C23^ii^	1.256 (4)
N1—C1	1.335 (4)	N3—C14	1.336 (4)
N1—C5	1.338 (4)	N3—C18	1.341 (4)
N2—C5	1.340 (4)	N4—C14	1.332 (5)
N2—H2A	0.8600	N4—H4A	0.8600
N2—H2B	0.8600	N4—H4B	0.8600
C1—C2	1.352 (5)	C14—C15	1.388 (5)
C1—H1	0.9300	C15—C16	1.327 (6)
C2—C3	1.381 (5)	C15—H14	0.9300
C2—H2	0.9300	C16—C17	1.382 (5)
C3—C4	1.349 (5)	C16—H15	0.9300
C3—H3	0.9300	C17—C18	1.339 (5)
C4—C5	1.398 (5)	C17—H16	0.9300
C4—H4	0.9300	C18—H18	0.9300
C6—C7	1.501 (5)	C19—C20	1.495 (4)
C7—C8	1.368 (6)	C20—C21	1.380 (5)
C7—C9	1.445 (5)	C20—C22	1.416 (5)
C8—H5	0.9300	C21—H21A	0.9300
C8—H6	0.9300	C21—H21B	0.9300
C9—H7	0.9600	C22—H22A	0.9600
C9—H8	0.9600	C22—H22B	0.9600
C9—H9	0.9600	C22—H22C	0.9600
C10—O8^i^	1.249 (4)	C23—O3^ii^	1.256 (4)
C10—C11	1.508 (4)	C23—C24	1.502 (5)
C11—C12	1.350 (6)	C24—C25	1.351 (5)
C11—C13	1.412 (6)	C24—C26	1.429 (6)
C12—H12A	0.9300	C25—H25A	0.9300
C12—H12B	0.9300	C25—H25B	0.9300
C13—H13A	0.9600	C26—H26A	0.9600
C13—H13B	0.9600	C26—H26B	0.9600
C13—H13C	0.9600	C26—H26C	0.9600
			
O7—Cu2—O8	167.76 (9)	O3—Cu1—O1^ii^	90.51 (9)
O7—Cu2—O6	88.23 (9)	O3—Cu1—O4	167.62 (10)
O8—Cu2—O6	89.74 (9)	O1^ii^—Cu1—O4	88.24 (10)
O7—Cu2—O5^i^	90.42 (10)	O3—Cu1—O2	89.23 (9)
O8—Cu2—O5^i^	88.94 (9)	O1^ii^—Cu1—O2	167.56 (9)
O6—Cu2—O5^i^	167.47 (10)	O4—Cu1—O2	89.34 (9)
O7—Cu2—N1	98.41 (9)	O3—Cu1—N3	93.68 (10)
O8—Cu2—N1	93.81 (9)	O1^ii^—Cu1—N3	100.03 (10)
O6—Cu2—N1	96.13 (10)	O4—Cu1—N3	98.67 (10)
O5^i^—Cu2—N1	96.39 (10)	O2—Cu1—N3	92.39 (9)
O7—Cu2—Cu2^i^	84.33 (7)	O3—Cu1—Cu1^ii^	83.37 (7)
O8—Cu2—Cu2^i^	83.48 (7)	O1^ii^—Cu1—Cu1^ii^	83.38 (7)
O6—Cu2—Cu2^i^	85.58 (7)	O4—Cu1—Cu1^ii^	84.24 (7)
O5^i^—Cu2—Cu2^i^	81.89 (7)	O2—Cu1—Cu1^ii^	84.24 (7)
N1—Cu2—Cu2^i^	176.80 (6)	N3—Cu1—Cu1^ii^	175.54 (8)
C6—O6—Cu2	121.8 (2)	C19—O2—Cu1	122.8 (2)
C6—O5—Cu2^i^	125.4 (2)	C19—O1—Cu1^ii^	124.1 (2)
C10—O7—Cu2	122.5 (2)	C23—O4—Cu1	122.9 (2)
C10^i^—O8—Cu2	123.3 (2)	C23^ii^—O3—Cu1	124.3 (2)
C1—N1—C5	116.5 (3)	C14—N3—C18	116.5 (3)
C1—N1—Cu2	115.9 (2)	C14—N3—Cu1	127.1 (3)
C5—N1—Cu2	127.5 (3)	C18—N3—Cu1	116.2 (2)
C5—N2—H2A	120.0	C14—N4—H4A	120.0
C5—N2—H2B	120.0	C14—N4—H4B	120.0
H2A—N2—H2B	120.0	H4A—N4—H4B	120.0
N1—C1—C2	125.6 (3)	N4—C14—N3	116.9 (4)
N1—C1—H1	117.2	N4—C14—C15	120.7 (4)
C2—C1—H1	117.2	N3—C14—C15	122.4 (4)
C1—C2—C3	116.9 (4)	C16—C15—C14	118.6 (4)
C1—C2—H2	121.5	C16—C15—H14	120.7
C3—C2—H2	121.5	C14—C15—H14	120.7
C4—C3—C2	120.2 (4)	C15—C16—C17	120.7 (4)
C4—C3—H3	119.9	C15—C16—H15	119.7
C2—C3—H3	119.9	C17—C16—H15	119.7
C3—C4—C5	119.0 (3)	C18—C17—C16	117.5 (4)
C3—C4—H4	120.5	C18—C17—H16	121.3
C5—C4—H4	120.5	C16—C17—H16	121.3
N1—C5—N2	117.6 (3)	C17—C18—N3	124.5 (4)
N1—C5—C4	121.8 (4)	C17—C18—H18	117.8
N2—C5—C4	120.6 (3)	N3—C18—H18	117.8
O6—C6—O5	125.1 (3)	O2—C19—O1	125.3 (3)
O6—C6—C7	117.6 (4)	O2—C19—C20	117.1 (3)
O5—C6—C7	117.2 (3)	O1—C19—C20	117.6 (3)
C8—C7—C9	125.1 (4)	C21—C20—C22	124.5 (3)
C8—C7—C6	117.7 (4)	C21—C20—C19	118.7 (3)
C9—C7—C6	117.2 (4)	C22—C20—C19	116.8 (3)
C7—C8—H5	120.0	C20—C21—H21A	120.0
C7—C8—H6	120.0	C20—C21—H21B	120.0
H5—C8—H6	120.0	H21A—C21—H21B	120.0
C7—C9—H7	109.5	C20—C22—H22A	109.5
C7—C9—H8	109.5	C20—C22—H22B	109.5
H7—C9—H8	109.5	H22A—C22—H22B	109.5
C7—C9—H9	109.5	C20—C22—H22C	109.5
H7—C9—H9	109.5	H22A—C22—H22C	109.5
H8—C9—H9	109.5	H22B—C22—H22C	109.5
O8^i^—C10—O7	126.2 (3)	O4—C23—O3^ii^	125.1 (3)
O8^i^—C10—C11	117.1 (3)	O4—C23—C24	117.2 (3)
O7—C10—C11	116.7 (3)	O3^ii^—C23—C24	117.6 (3)
C12—C11—C13	123.1 (3)	C25—C24—C26	122.6 (4)
C12—C11—C10	119.4 (4)	C25—C24—C23	120.0 (4)
C13—C11—C10	117.4 (4)	C26—C24—C23	117.4 (4)
C11—C12—H12A	120.0	C24—C25—H25A	120.0
C11—C12—H12B	120.0	C24—C25—H25B	120.0
H12A—C12—H12B	120.0	H25A—C25—H25B	120.0
C11—C13—H13A	109.5	C24—C26—H26A	109.5
C11—C13—H13B	109.5	C24—C26—H26B	109.5
H13A—C13—H13B	109.5	H26A—C26—H26B	109.5
C11—C13—H13C	109.5	C24—C26—H26C	109.5
H13A—C13—H13C	109.5	H26A—C26—H26C	109.5
H13B—C13—H13C	109.5	H26B—C26—H26C	109.5

Symmetry codes: (i) −*x*+1, −*y*, −*z*+1; (ii) −*x*+2, −*y*+1, −*z*+2.

### Hydrogen-bond geometry (Å, °)

**Table:**

*D*—H···*A*	*D*—H	H···*A*	*D*···*A*	*D*—H···*A*
N2—H2A···O5^i^	0.86	2.23	2.963 (4)	144
N2—H2B···O2^iii^	0.86	2.58	3.334 (3)	148
N4—H4A···O4	0.86	2.30	3.051 (4)	145

Symmetry codes: (i) −*x*+1, −*y*, −*z*+1; (iii) −*x*+1, *y*−1/2, −*z*+3/2.
